# Successful Spontaneous Pregnancy and Live Birth in a Woman With Premature Ovarian Insufficiency and 10 Years of Amenorrhea: A Case Report

**DOI:** 10.3389/fmed.2020.00018

**Published:** 2020-02-07

**Authors:** Ying Gu, Yan Xu

**Affiliations:** ^1^Department of Urology and Reproductive Medicine, Seventh People's Hospital of Shanghai University of Traditional Chinese Medicine, Shanghai, China; ^2^Department of Gynaecology and Obstetrics, Seventh People's Hospital of Shanghai University of Traditional Chinese Medicine, Shanghai, China

**Keywords:** premature ovarian failure, spontaneous pregnancy, amenorrhea, live birth, hormone replacement therapy

## Abstract

**Background:** Primary ovarian insufficiency (POI) is a devastating diagnosis for reproductive-aged women due to the associated infertility and other serious health consequences. Spontaneous pregnancy without hormone replacement therapy (HRT) and/or assisted reproductive technology (ART) rarely occurs in POI patients, particularly in those patients with long-term amenorrhea.

**Case:** On March 4, 2019, a 31-year-old Chinese POI patient visited our hospital for a spontaneous pregnancy after 10 years of amenorrhea and discontinuation of HRT 4 years prior. The patient had menarche at the age of 13, with 3 years of regular menstruation followed by amenorrhea occurring at the age of 20. POI was diagnosed by several hospitals; chromosome examination revealed a normal 46, XX karyotype. Treatment with estradiol valerate and progesterone did lead to resumed menstruation, while amenorrhea resumed after withdrawal of HRT. The patient married at the age of 23 and tried to conceive by HRT until the age of 25; her beta-human chorionic gonadotropin (HCG), estrogen (E2), and progesterone levels were 32987.7~119151.4 mIU/ml, 671.0~>1,000 pg/mL, and 6.6~27.9 ng/ml, respectively. On March 22, 2019, ultrasonography showed an intrauterine pregnancy with a normally developed gestational sac sized 45 × 42 × 32 mm with a 17 mm crown-rump length. On October 29, a 3,400 g healthy girl baby was delivered; the patient had a spontaneous delivery with natural labor.

**Conclusion:** Spontaneous pregnancy is possible in women with POI and 10 years of amenorrhea.

## Background

Premature ovarian failure (POF) refers to amenorrhea before the age of 40 caused by ovarian failure; POF is characterized by primary or secondary amenorrhea with elevated serum gonadotropin levels and decreased estrogen levels and is often accompanied by a series of low estrogen symptoms, such as hot flushes, excessive sweating, hair loss, skin and mucous membrane dryness, and low sexual desire, which vary in degrees among individuals (1). Currently, POI is the preferred term advocated by the National Institutes of Health; it includes previous terms of premature menopause or POF, because ovarian function is intermittent or unpredictable in many cases (1, 2). Lower fertility or even infertility is the most disturbing POI-related issue for women of childbearing age (1–3). Although reports have shown that ~5–10% of women conceive and deliver a child after they have received the diagnosis of POI (1–3), this term describes a spectrum of declining ovarian function and reduced fecundity due to a premature decrease in initial follicle number, an increase in follicle destruction, or poor follicular response to gonadotropins (1–4). Thus, the rate of spontaneous pregnancy differs by pathological condition.

## Case Presentation

A 31-year-old female with no family history of infertility, POI, or autoimmune disease visited our hospital for a long-desired early pregnancy. The patient denied a history of chemotherapy or radiation therapy. The patient had menarche at the age of 13, with regular menstruation until the age of 17; then, disordered menstruation emerged, and amenorrhea occurred at the age of 20. POI was diagnosed by several hospitals; chromosomal analysis demonstrated a normal female karyotype (46, XX); treatment with estradiol valerate and progesterone led to menstruation, while amenorrhea continued after withdrawal of HRT. The patient married at the age of 23 and had tried to conceive by HRT until the age of 25 (at which point she had given up after failing to conceive); her last menstruation was July 2014; amenorrhea continued to date. At the age of 29, because of her yearning for fertility, the patient visited a general hospital in Shanghai. Clinical tests showed luteinizing hormone (LH), 23.9 IU/L; follicle-stimulating hormone (FSH), 69.1 IU/L; estrogen, 43.3 pg/mL; and testosterone, 1.1 nmol/L. Infertility and POI were again confirmed. No sinus follicles were found in either ovary under ultrasonography. The patient lived a normal couple's life after marriage. On March 4, 2019, the patient visited our department due to nausea, vomiting, a small amount of vaginal bleeding, and a positive urinary pregnancy test. Examinations showed a normal physique for women of childbearing age, normally developed breasts, a normal distribution of pubic hair, vaginal patency, normal cervical development, and a uterus size consistent with 6+ weeks of pregnancy.

Ultrasonography showed an intrauterine early pregnancy with an insufficiently filled gestational sac sized 23 × 20 × 11 mm; the yolk sac, punctate embryo tissue and heart tube pulsation could be seen. HCG, estrogen, and progesterone were 32987.7~119151.4 mIU/ml, 671.0~>1,000 pg/mL, and 6.6~27.9 ng/ml, respectively. Anti-Müllerian hormone (AMH) was 0.08 ng/ml. Considering the threat of spontaneous abortion, POI and insufficient luteal function, luteum support therapy was adopted. The treatment regimen included intramuscular injection of progesterone (40 mg, QD, 15 days), dydrogesterone (10 mg BID orally for 45 days), and estradiol valerate (4 mg BID orally for 15 days). On March 8, 2019, the vaginal bleeding stopped. On March 22, 2019, ultrasonography showed a normally developed gestational sac sized 45 × 42 × 32 mm and a 17 mm crown-rump length; the yolk sac, punctate embryo tissue, and heart tube pulsation could be seen. The patient was in normal progress of a full-term pregnancy. On October 29, a 3,400 g healthy girl baby was delivered; the patient had a spontaneous delivery with natural labor.

## Discussion

The incidence of POI is 1 in 1,000 women younger than 30 years of age (3). Although the literature states that 5–10% of women with POI could conceive and deliver a child (1–4), this ratio lacks confirmation in large-scale population studies; in addition, since POI or POF describes a spectrum of declining oocyte quality, oocyte quantity, or reproductive potential with different outcomes, the rate of spontaneous pregnancy differs by pathological conditions. Successful spontaneous pregnancy occurred in this POI case after 10 years of amenorrhea, emphasizing the real chances for spontaneous conception and intermittent and unpredictable ovarian function in these patients. The main features of our cases were as follows: a 31-year-old female with 10 years of amenorrhea spontaneously conceived and delivered a healthy baby without ART; there are very few similar cases available for reference. Almost all accessible case reports described women who achieved pregnancy under intervention, including HRT (5–8); ovulation induction (9); donor egg *in vitro* fertilization (10); ovariectomy for ovarian tissue cryopreservation, followed by *in vitro* activation (IVA) for infertility treatment (11); immediate ovarian stimulation and *in vitro* fertilization (12); drug-free *in vitro* activation of follicles and fresh tissue autotransplantation (13), and autologous grafting of cryopreserved ovarian cortex at an orthotopic site (14), etc. A 29-year-old Indian woman with primary amenorrhea characterized by a few episodes of withdrawal bleeding with HRT in the past was considered to be POI; she completed a full-term pregnancy without any assisted reproductive measures, however, the authors did not show the duration of amenorrhea and the FSH level, so the POI described lacks diagnostic evidence (15). Spontaneous pregnancy in a 33-year-old Mexican woman with POF was reported; however, we could not learn the details of this patient (16). Spontaneous pregnancy occurred in a 37-year-old POF female 9 months after a previous full-term pregnancy following assisted reproduction with oocyte donation; and we could not exclude the residual impacts of the HRT (17).

The etiology of POI includes genetic predisposition (18), autoimmune and enzymatic disorders (12), infections (4), and iatrogenic causes (4); however, in many cases, the etiology of POI remains unknown because POI describes a spectrum of declining ovarian function and reduced fecundity due to a premature decrease in the initial follicle number, an increase in follicle destruction, or poor follicular response to gonadotropins (1–4). Although no abnormality was found by chromosome examination in this case, we did not perform tests for fragile X mental retardation 1 (FMR1) and autoimmune regulator (AIRE) genes, which is one of the shortcomings of our report. An interesting case from China showed that POI was induced by treatment with tripterygium glycosides in a woman with nephrotic syndrome (9); our case denied any treatment with traditional Chinese medicine. It is difficult to speculate the etiology of this case; a live birth derived from spontaneous pregnancy negated many of the pathological conditions.

The American College of Obstetricians and Gynecologists and the American Society for Reproductive Medicine recommended performing two random tests of FSH and estradiol levels at least 1 month apart because a single value has very limited reliability due to high inter- and intracycle variability, particularly if FSH is not elevated (1, 19). In this report, the diagnosis of POI was based on the following clinical characteristics: (1) 10 years of amenorrhea; (2) high basal serum FSH concentration (69.1 IU/L, although only one test); (3) low basal estrogen level, 43.3 pg/mL; (4) low AMH level, 0.08 ng/ml; and (5) no sinus follicles were found in both ovaries under ultrasonography. Since there is currently no consensus on criteria to identify POI in adolescents, in clinical practice, the diagnosis of POI is accurate. Our case is a typical case to show that ovarian function is intermittent or unpredictable. First, successful ovulation, conception and delivery occurred. Second, initial ultrasonography showed a gestational sac sized 23 × 20 × 11 mm, which is equivalent to 6–7 weeks of pregnancy. HCG, estrogen, and progesterone measured at the same time point were 32987.7 mIU/ml, 671.0 pg/mL, and 6.6 ng/ml, respectively. Although the HCG level was a bit higher and progesterone level was much lower than the corresponding levels for the same gestational age (which still indicates ovarian insufficiency of this patient), the estrogen level indicated that the ovary had some level endocrine function in the absence of intervention. In addition, progesterone was as low as 6.6 ng/ml at the first outpatient visit, and a timely treatment regimen including intramuscular injection of progesterone (40 mg, QD) should be a beneficial intervention in similar case.

## Data Availability Statement

All datasets generated for this study are included in the article/supplementary material.

## Ethics Statement

This study was approved by the Ethical Review Committee of the Shanghai 7th People's Hospital in accordance with the World Medical Association Declaration of Helsinki. Written informed consents (mother and her baby) were obtained from the participant (mother) for the publication of this case report. The patient was informed of her right to withdraw consent personally or via kin, a caretaker, or a guardian.

## Author Contributions

YG researched the literature and drafted the manuscript. YX reviewed and revised the manuscript and obtained patient consent.

### Conflict of Interest

The authors declare that the research was conducted in the absence of any commercial or financial relationships that could be construed as a potential conflict of interest.
